# Evaluation of pregnancy outcomes in patients with a history of bariatric surgery

**DOI:** 10.3389/fmed.2025.1609344

**Published:** 2025-06-11

**Authors:** Ufuk Atlihan, Onur Yavuz, Can Ata, Huseyin Aytug Avsar, Selcuk Erkilinc

**Affiliations:** ^1^Department of Obstetrics and Gynecology, Merkezefendi Hospital, Manisa, Türkiye; ^2^Department of Obstetrics and Gynecology, Dokuz Eylul University School of Medicine, Izmir, Türkiye; ^3^Department of Obstetrics and Gynecology, Buca Seyfi Demirsoy Training and Research Hospital, Izmir Democracy University, Izmir, Türkiye; ^4^Department of Obstetrics and Gynecology, Tinaztepe University School of Medicine Galen Hospital, Izmir, Türkiye

**Keywords:** bariatric surgery, high-risk pregnancy, neonatal outcomes, pregnancy outcomes, obesity

## Abstract

**Introduction:**

Obesity and overweight are significant risk factors for perinatal morbidity and mortality, and an increasing number of women of reproductive age are being offered bariatric surgery. The present study investigated the outcomes of pregnancies and births after bariatric surgery (BS).

**Materials and methods:**

All patients who gave birth at our clinic between 2018 and 2023 were included in the study. Individuals who had undergone BS previously were identified using the hospital database, and their medical birth records were obtained for data on pregnancy, delivery, and perinatal results. The results of women who had undergone BS previously were analyzed by comparing them with other pregnancies.

**Results:**

A total of 298 women who had undergone BS previously and 4,374 women who had not undergone surgeries were included in the study. The BS group had higher rates of abortion (*p* = 0.009) and IVF history (*p* < 0.001). Additionally, the incidence of pregnancy-induced hypertension (*p* < 0.001), preeclampsia (*p* = 0.04), gestational diabetes (GDM) (*p* < 0.001), premature birth (*p* < 0.001), cesarean delivery (*p* < 0.001), and small for gestational age (*p* < 0.001) was significantly higher in the BS group.

**Conclusion:**

Given the potential hazards associated with obesity in women of reproductive age, BS may be seen as a prudent course of therapy. Our study concluded that pregnancy prognosis is closely correlated with BMI at the time of pregnancy. Previous research revealed similar findings between groups in terms of pregnancy prognosis and pregnancy complications in obese and extremely obese patient groups.

## Introduction

The rate of women of reproductive age with overweight and obesity has risen to unprecedented levels (1). Obesity among women in their reproductive years has grown in the United States and many other industrialized nations in recent years (1). Obesity has been rising globally for decades and is linked to an increased incidence of cardiometabolic comorbidities and mortality (1). Effective weight loss techniques are needed to end the long-term harmful effects of obesity. Obesity and being overweight raise the risk of unfavorable pregnancy and delivery outcomes and are significant risk factors for perinatal morbidity and death (2), with negative effects on fertility and fetal development (2).

As a lifestyle change and medical treatment, bariatric surgery (BS) is widely employed in individuals with extreme obesity and has been proven to yield good weight loss results (3). The number of obesity surgeries has increased by 800% in recent years, with positive long-term results obtained after BS (3). For this reason, BS is acknowledged as the most efficient treatment modality for extreme obesity (4). It is already known as the only therapeutic approach that helps people with obesity lose weight in a significant and long-lasting way (5, 6). On a global scale, laparoscopic BS is now the most common BS technique (7). Although BS is considered to be very safe, there remains doubt about the hazards involved with becoming pregnant and giving birth after undergoing BS surgery (3, 8). It is very important to determine the benefits and hazards of this surgery regarding births, future pregnancies, and fertility because women of reproductive age also undergo BS (9).

Although previous studies reported that pregnancy outcomes were generally improved after BS, a higher risk of small for gestational age (SGA) babies and premature birth was also reported (10–12). After BS, pregnant women must be regularly checked by a specialist group of experts from different areas with experience in management after BS procedures. The special requirements of this patient group during pregnancy must be dealt with individually (13).

The present study aimed to evaluate the effects of bariatric surgery on pregnancy, delivery, and neonatal outcomes in women who became pregnant after BS.

## Materials and method

This research had a retrospective multicenter cohort study design and followed the Declaration of Helsinki Principles. The study was started after receiving ethics committee approval from our hospital (Date: 28/02/24, Number: 2024/249). The data of 298 individuals with a BS history and 4,374 patients without a BS history, whose pregnancy follow-ups and births were performed by us between March 2018 and March 2023, were evaluated retrospectively from patient files and the hospital database. In our study, all patients underwent sleeve gastrectomy using the BS method. The mean time between BS and pregnancy was determined as 20 months (range 14–33 months) for the patients in the study cohort. Considering the time elapsed after the surgery, patients with weight stability at the beginning of pregnancy were included in the evaluation. The age, body mass index (BMI), smoking history during pregnancy, parity, abortion, and *in vitro* fertilization (IVF) history of the patients were evaluated retrospectively. The data regarding pregnancy-related problems, pregnancy-induced hypertension (PIH), preeclampsia, premature birth history, gestational diabetes (GDM), insulin-dependent GDM, and type of birth were analyzed. Large for gestational age (LGA), small for gestational age (SGA), and 5th-minute Apgar scores were evaluated. Chronic hypertension, diabetes mellitus, the presence of systemic and autoimmune diseases, and multiple pregnancies were considered as exclusion criteria. The American Diabetes Association Criteria were used to diagnose gestational diabetes (14). Pregnancy-induced hypertension was diagnosed in accordance with the most recent American College of Obstetricians and Gynecologists bulletin (15). Participants were further categorized into four groups based on their BMI to further evaluate individual results following BS: Normal weight (BMI ≤ 24.9 kg/m^2^), Overweight (BMI 25.0–30 kg/m^2^), Obese (BMI 30–34.9 kg/m^2^), and Extremely obese (BMI > 35.0 kg/m^2^).

### Statistical analysis

Statistical analysis was performed using the SPSS Ver. 26.0 software package (IBM Inc., Chicago, IL, USA). The Kolmogorov–Smirnov test was used to assess the normality of data distribution. The parameters that were not normally distributed were examined using the Mann–Whitney U-test. For the examination of categorical data, Fisher’s exact test and the chi-squared test were used. The characteristics that were not normally distributed are presented as median (minimum-maximum). Numerical and percentage (%) statistics were used to represent qualitative data and 95% confidence intervals (CIs) were used to analyze the results. *p*-values of less than 0.05 were regarded as statistically significant.

## Results

The incidence of miscarriages (≥1) was 24.5% in individuals who had undergone BS, compared to 18.4% in those who had not, showing statistical significance (*p* = 0.009). The IVF rate among participants who underwent BS was significantly higher than that of patients who did not undergo BS (5.7% vs. 2.1%, respectively) (*p* < 0.001). The PIH rate was statistically significantly higher in the BS group than in those who did not undergo BS (6.7% vs. 3.2%, respectively) (*p* < 0.001). Similarly, the preeclampsia rate was higher in the BS group (2.3%) than in those who did not undergo BS (1.1%) (*p* = 0.04). The GDM rate was significantly higher (14.8%) in participants who underwent BS than those who did not undergo BS (8.2%) (*p* < 0.001). Insulin treatment for GDM was required in 4.4% of participants who underwent BS, whereas it was 1.1% in those who did not undergo BS, reflecting a significant difference (*p* < 0.001). Premature delivery rates were higher in the BS group than in those who did not undergo BS (8.7% vs. 4.4%, respectively) (*p* < 0.001). The vaginal birth rate was lower in the BS group (64.8%) than in the non-BS group (74.8%) (*p* < 0.001). The C/S rate was higher in participants who underwent BS (35.2%) than those who did not undergo BS (25.1%) (*p* < 0.001). The SGA rate was higher in the BS group (8.7%) than in those who did not undergo BS (3.2%) (*p* < 0.001). Finally, the rate of participants with Apgar ≤7 (5 min) scores was higher in the BS group (7.4%) than in those who did not undergo BS (1.9%) (*p* < 0.001) (Table 1).

**Table 1:** Comparison of the demographic characteristics of the groups that did and did not undergo BS.

Variables	Bariatric group (*n* = 298, 6.4%)	Non-Bariatric group (*n* = 4,374, 93.6%)	*p*-value
Age (years)	29 (23–43)	31 (21–42)	0.1
BMI (kg/m^2^)	24.8 (19.1–39.8)	24.8 (18.6–39.9)	0.1
BMI subgroups (kg/m^2^)			
<25%	50.7% (151/298)	51.9% (2,272/4374)	
25–29.9%	28.2% (84/298)	30.9% (1,350/4374)	0.3
30–34.9%	14.1% (42/298)	11.3% (494/4374)	
≥ 35%	7% (21/298)	5.9% (258/4374)	
Smoking during pregnancy	20.1% (60/298)	17.2% (751/4374)	0.1
Primiparity	53.7% (160/298)	59.2% (2,591/4374)	0.06
Multiparity (>3 deliveries)	9.7% (29/298)	10.7% (469/4374)	0.5
Miscarriages ( ≥ 1)	24.5% (73/298)	18.4% (804/4374)	0.009
IVF	5.7% (17/298)	2.1% (90/4374)	<0.001
PIH	6.7% (20/298)	3.2% (141/4374)	<0.001
Preeclampsia	2.3% (7/298)	1.1% (47/4374)	0.04
GDM	14.8% (44/298)	8.2% (358/4374)	<0.001
Insulin treatment for GDM	4.4% (13/298)	1.5% (65/4374)	<0.001
Premature delivery (<37 weeks)	8.7% (26/298)	4.4% (191/4374)	<0.001
Postterm delivery (>42 weeks)	2% (6/298)	2.6% (114/4374)	0.5
Vaginal birth	64.8% (193/298)	74.8% (3,271/4374)	<0.001
C/S	35.2% (105/298)	25.1% (1,096/4374)	<0.001
Stillbirth	0% (0/298)	0.2% (7/4374)	0.4
SGA	8.7% (26/298)	3.2% (139/4374)	<0.001
LGA	1.3% (4/298)	2.2% (95/4374)	0.3
Apgar ≤ 7 (5 min)	7.4% (22/298)	1.9% (83/4374)	<0.001

BMI, Body mass index; IVF, In vitro fertilization; PIH, Pregnancy-induced hypertension; GDM, Gestational diabetes mellitus; C/S, Cesarean section; SGA, Small for gestational age; LGA, Large for gestational age.

When comparing patient groups with a BMI < 25 kg/m^2^, the rate of PIH among participants who underwent BS was statistically significantly higher at 9.9% than those who did not undergo BS, which had a rate of 3.5% (*p* < 0.001). The rate of preeclampsia in participants who underwent BS was also statistically significantly higher than in those who did not undergo BS, at 4.6% vs. 1.1%, respectively (*p* < 0.001). Furthermore, the rate of GDM among participants who underwent BS was statistically significantly higher than those who did not undergo BS, at 17.9% vs. 8.8%, respectively (*p* < 0.001). The rate of insulin treatment for GDM among participants who underwent BS was 5.3%, compared to 1.7% in those who did not undergo BS, with a statistically significantly higher incidence in the BS group (*p* = 0.002). The premature delivery rate was 13.2% for participants who underwent BS, versus 4.8% for those who did not, indicating a statistically significant increase in the BS group (*p* < 0.001). Vaginal birth rates were lower at 64.9% in the BS group, compared to 74.1% in the non-BS group, demonstrating statistical significance (*p* = 0.01). Conversely, the C/S rate was higher in the BS group at 35.1%, compared to 25.7% in the non-BS group, also showing statistical significance (*p* = 0.01). SGA rates were significantly elevated in the BS group at 13.2%, as opposed to 3.4% in the non-BS group (*p* < 0.001). Additionally, the incidence of Apgar scores ≤7 at 5 min was higher in the BS group at 9.9%, compared to 1.9% in the non-BS cohort, with statistically significant differences (*p* < 0.001) (Table 2).

**Table 2:** The comparison of the-demographic characteristics of the groups (BMI <25 kg/m^2^) that did and did not undergo BS.

Variables	Bariatric group (*n* = 151, 6.2%)	Non-Bariatric group (*n* = 2,272, 93.8%)	*p*-value
Age (years)	30 (23–43)	31 (22–42)	0.2
BMI (kg/m^2^)	22.6 (19.1–24.6)	22.5 (18.6–24.9)	0.1
Smoking during pregnancy	17.9% (27/151)	17% (386/2272)	0.7
Primiparity	53% (80/151)	59.2% (1,344/2272)	0.1
Multiparity (>3 deliveries)	13.2% (20/151)	11% (249/2272)	0.3
Miscarriages (1)	23.2% (35/151)	18.4% (419/2272)	0.1
IVF	2.6% (4/151)	2% (46/2272)	0.6
PIH	9.9% (15/151)	3.5% (79/2272)	<0.001
Preeclampsia	4.6% (7/151)	1.1% (26/2272)	<0.001
GDM	17.9% (27/151)	8.8% (200/2272)	<0.001
Insulin treatment for GDM	5.3% (8/151)	1.7% (39/2272)	0.002
Premature delivery (<37 weeks)	13.2% (20/151)	4.8% (108/2272)	<0.001
Postterm delivery (>42 weeks)	3.3% (5/151)	2.9% (67/2272)	0.8
Vaginal birth	64.9% (98/151)	74.1% (1,684/2272)	0.01
C/S	35.1% (53/151)	25.7% (584/2272)	0.01
Stillbirth	0% (0/151)	0.1% (3/2272)	0.6
SGA	13.2% (20/151)	3.4% (77/2272)	<0.001
LGA	2% (3/151)	2.1% (47/2272)	0.9
Apgar 7 (5 min.)	9.9% (15/151)	1.9% (44/2272)	<0.001

BMI, Body mass index; IVF, In vitro fertilization; PIH, Pregnancy-induced hypertension; GDM, Gestational diabetes mellitus; C/S, Cesarean section; SGA, Small for gestational age; LGA, Large for gestational age.

In the participant group with BMI (25–29.9) kg/m^2^, the mean age of those who underwent BS was 28, whereas it was 31 years in those who did not undergo BS, showing a significantly lower mean age in the BS group (*p* < 0.001). The rate of miscarriages (≥1) among participants who had BS was 27.4%, compared to 17.9% in those who did not have BS, which was statistically higher in the BS group (*p* = 0.03). The IVF rate for participants who underwent BS was 13.1%, whereas it was 1.7% for those who did not, also statistically higher in the BS group (*p* < 0.001).

The rate of GDM in participants who had BS was 14.3%, compared to 7.5% in those who did not have BS, again statistically higher in the BS group (*p* = 0.02). Insulin treatment for GDM was required for 4.8% of participants who had BS, compared to 1.3% for those who did not, with the BS group showing a statistically higher rate (*p* = 0.01). The vaginal birth rate for participants who had BS was 59.5%, whereas it was 74.9% for those who did not undergo BS, which was statistically lower in the BS group (*p* = 0.002). The C/S rate for participants who had BS was 40.5%, as opposed to 25% for those who did not, making it statistically higher in the BS group (*p* = 0.002). Finally, the rate of Apgar ≤7 (5 min) scores was 6% for participants who had BS, compared to 1.9% for those who did not, statistically higher in the BS group (*p* = 0.01) (Table 3).

**Table 3:** Comparison of the demographic characteristics of groups (BMI 25–29.9 kg/m^2^) that did and did not undergo BS.

Variables	Bariatric group (*n* = 84, 5.9%)	Non-Bariatric group (*n* = 1,350, 94.1%)	*p*-value
Age (years)	28 (23–42)	31 (22–42)	<0.001
BMI (kg/m^2^)	26.8i(25.1–28.7)	26.7 (25.1–29.8)	0.5
Smoking during pregnancy	17.9% (15/84)	17.5% (236/1350)	0.9
Primiparity	53.6% (45/84)	57.7% (781/1350)	0.4
Multiparity (>3 deliveries)	6% (5/84)	10.9% (147/1350)	0.1
Miscarriages ( ≥ 1)	27.4% (23/84)	17.9% (242/1350)	0.03
IVF	13.1% (11/84)	1.7% (23/1350)	<0.001
PIH	1.2% (1/84)	2.4% (33/1350)	0.4
Preeclampsia	0% (0/84)	0.8% (11/1350)	0.4
GDM	14.3% (12/84)	7.5% (101/1350)	0.02
Insulin treatment for GDM	4.8% (4/84)	1.3% (18/1350)	0.01
Premature delivery (<37 weeks)	4.8% (4/84)	4% (54/1350)	0.7
Postterm delivery (>42 weeks)	1.2% (1/84)	2.4% (33/1350)	0.4
Vaginal birth	59.5% (50/84)	74.9% (1,011/1350)	0.002
C/S	40.5% (34/84)	25% (337/1350)	0.002
Stillbirth	0% (0/84)	0.1% (1/1350)	0.8
SGA	3.6% (3/84)	3.2% (43/1350)	0.8
LGA	1.2% (1/84)	2.4% (32/1350)	0.4
Apgar ≤ 7 (5 min.)	6% (5/84)	1.9% (26/1350)	0.01

BMI, Body mass index; IVF, In vitro fertilization; PIH, Pregnancy-induced hypertension; GDM, Gestational diabetes mellitus; C/S, Cesarean section; SGA, Small for gestational age; LGA, Large for gestational age.

Among participants with a BMI of 30–34.9 kg/m^2^, the mean age was 28 years for those who had BS and 31 for those who did not, with a significantly lower mean age in the BS group (*p* < 0.001). The smoking rate during pregnancy was 35.7% for participants who had undergone BS and 17.6% for those who had not, with a significantly higher rate in the BS group (*p* = 0.004) (Table 4).

**Table 4:** The comparison of the demographic characteristics of groups (BMI 30–34.9 kg/m^2^) that underwent and did not undergo BS.

Variables	Bariatric group (*n* = 42, 7.8%)	Non-Bariatric group (*n* = 494, 92.2%)	*p*-value
Age (years)	28 (23–37)	31 (22–42)	<0.001
BMI (%)	33 (30.6–34.7)	32.6 (30.1–34.9)	0.01
Smoking during pregnancy	35.7% (15/42)	17.6% (87/494)	0.004
Primiparity	54.8% (23/42)	60.7% (300/494)	0.4
Multiparity (>3 deliveries)	4.8% (2/42)	10.5% (52/494)	0.2
Miscarriages (1)	28.6% (12/42)	20.2% (100/494)	0.2
IVF	4.8% (2/42)	2.8% (14/494)	0.4
PIH	4.8% (2/42)	3.8% (19/494)	0.7
Preeclampsia	0% (0/42)	1.4% (7/494)	0.4
GDM	9.5% (4/42)	7.9% (39/494)	0.7
Insulin treatment for GDM	2.4% (1/42)	1% (5/494)	0.4
Premature delivery (<37 weeks)	4.8% (2/42)	4.3% (21/494)	0.8
Postterm delivery (>42 weeks)	0% (0/42)	2.2% (11/494)	0.3
Vaginal birth	73.8% (31/42)	75.7% (374/494)	0.7
C/S	26.2% (11/42)	24.1% (119/494)	0.7
Stillbirth	0% (0/42)	0.4% (2/494)	0.6
SGA	7.1% (3/42)	2.6% (13/494)	0.09
LGA	0% (0/42)	2.4% (12/494)	0.3
Apgar 7 (5 min.)	2.4% (1/42)	1.8% (9/494)	0.7

BMI, Body mass index; IVF, In vitro fertilization; PIH, Pregnancy-induced hypertension; GDM, Gestational diabetes mellitus; C/S, Cesarean section; SGA, Small for gestational age; LGA, Large for gestational age.

In participants with a BMI over 35 kg/m^2^, the mean age was 28 years for those who had BS and 31 years for those who did not. The BS group had a significantly lower mean age (*p* = 0.03) (Table 5).

**Table 5:** Comparison of the demographic characteristics of groups (BMI >35 kg/m^2^) that did and did not undergo BS.

Variables	Bariatric group (*n* = 21, 7.5%)	Non-Bariatric group (*n* = 258, 92.5%)	*p*-value
Age (years)	28 (24–38)	31 (21–40)	0.03
BMI (kg/m^2^)	37 (36.1–39.8)	37 (35.1–39.9)	0.9
Smoking during pregnancy	14.3% (3/21)	16.3% (42/258)	0.8
Primiparity	57.1% (12/21)	64.3% (166/258)	0.5
Multiparity (>3 deliveries)	9.5% (2/21)	8.1% (21/258)	0.8
Miscarriages (1)	14.3% (3/21)	16.7% (43/258)	0.7
IVF	0% (0/21)	2.7% (7/258)	0.4
PIH	9.5% (2/21)	3.9% (10/258)	0.2
Preeclampsia	0% (0/21)	1.2% (3/258)	0.6
GDM	4.8% (1/21)	7% (18/258)	0.6
Insulin treatment for GDM	0% (0/21)	1.2% (3/258)	0.6
Premature delivery (<37 weeks)	0% (0/21)	3.1% (8/258)	0.4
Postterm delivery (>42 weeks)	0% (0/21)	1.1% (3/258)	0.6
Vaginal birth	66.7% (14/21)	78.3% (202/258)	0.2
C/S	33.3% (7/21)	21.7% (56/258)	0.2
Stillbirth	0% (0/21)	0.4% (1/258)	0.7
SGA	0% (0/21)	2.3% (6/258)	0.4
LGA	0% (0/21)	1.6% (4/258)	0.5
Apgar 7 (5 min.)	4.8% (1/21)	1.6% (4/258)	0.2

BMI, Body mass index; IVF, In vitro fertilization; PIH, Pregnancy-induced hypertension; GDM, Gestational diabetes mellitus; C/S, Cesarean section; SGA, Small for gestational age; LGA, Large for gestational age.

## Discussion

The results of the present research give a good overview of pregnancies and births after BS, showing that there is an elevation in the frequency of PIH, GDM, insulin-dependent GDM, abortion, preeclampsia, C/S, IVF, SGA and premature deliveries in women who undergo BS, with regard to controls without BS. A statistically significant difference in obstetric complications emerged in the data of the general study cohort without BMI-dependent matching between the groups. However, when BMI-matched subgroups were evaluated, it was noted that the main parameter revealing the difference between the groups was BMI. No difference was found between obstetric complications in the obese and extremely obese groups evaluated as BMI 30–34.9 kg/m^2^ and >35 kg/m^2^. However, in the normal weight and slightly overweight groups evaluated as BMI < 25 kg/m^2^ and 25–29.9 kg/m^2^, a statistically significant increase in obstetric complications was detected in the group that had undergone BS. The reason for this may be secondary to the effects of chronic inflammation caused by obesity experienced by patients undergoing BS in the current BMI subgroups and possible gastrointestinal absorption defects after the surgery.

No significant differences were detected between the groups on the basis of large for gestational age, smoking history, and parity. In the present study, comparisons made independently of BMI measurements demonstrated no significant differences in the mean age of the participants who had undergone BS and those who had not. The mean age of the participants who had BS in the slightly overweight, obese, and severely obese groups and BMI-matched patient groups was observed to be significantly lower than in the participants who did not have surgery. In the meta-analysis conducted by Akhter et al., the mean age was reported to be significantly higher in women who had undergone BS (16). When BMI-specific groups were compared, no significant differences were detected between the groups based on primiparity and multiparity. In the research of Stephansson et al., it was reported that women in the BS group gave birth more (11). In our study, no significant differences were detected between the average BMI of women who had undergone BS and the women in the other group. In the study of Kushner et al., residual obesity and even weight gain were detected several years after BS (17).

People with obesity are more likely to smoke (18), and smoking has been shown to increase the risk of metabolic syndrome and diabetes (19). In our study, the rate of smoking was significantly higher in the BS group among women with a BMI of 30–34.9 kg/m^2^; no significant difference was found in the other groups. In the study conducted by Stephansson et al., the rate of smoking was found to be higher in the group with a history of BS (11). However, a probable correlation between low weight and smoking after BS is difficult to demonstrate because bias cannot be ruled out since the pattern and frequency of smoking are reported by smokers.

Improved prognosis in pregnancies after BS was described previously (20). It is reasonable to compare pregnancies before and after BS (21). One crucial topic to consider is whether weight following BS has any bearing on prognosis and if there are risks associated with the surgery. Although studies are reporting that BS has positive impacts on fertility, increasing age may affect the results negatively (22, 23). In the study by Musella et al., BMI was reported as the only factor determining fertility after BS (22). In the present research, it was detected that IVF rates rise significantly in women after BS. Conflicting results were obtained in our study between IVF rates in BMI-matched groups. Although the results of IVF treatments were not evaluated in our study, obesity is predicted to have a detrimental impact on these outcomes. Our results also showed that women who had undergone BS had a higher abortion rate. Nevertheless, there are conflicting results regarding abortion rates in different BMI groups. The relationship between BS and abortion was not evaluated in a previous study conducted by Poston et al., and it was assumed that it was associated with obesity itself (2).

There are important studies reporting the correlation between obesity and the development of preeclampsia (24). Research has also shown that the risk of hypertensive disorder is increased in patients with obesity and extreme obesity (2, 25). In another study by Bennett et al., the incidence of preeclampsia was demonstrated to be significantly higher in individuals who had undergone BS (26). There is also strong evidence in the literature showing that the incidence of preeclampsia is reduced after BS (27, 28). It was reported in a meta-analysis by Galazis et al. that BS was associated with decreased preeclampsia rates and that this risk was reduced by approximately half (10). In our study, the rate of preeclampsia was detected to be significantly higher in individuals who had undergone BS when compared to those who had not. However, although the preeclampsia rates were significantly higher for normal-weight participant subgroups and the participants who had undergone BS, no significant differences were detected between the groups on the basis of preeclampsia rates in patients with obesity and extreme obesity.

Our study also demonstrated a higher incidence of PIH in the BS group; however, no differences were observed among patients with overweight, obesity, and extreme obesity in BMI-specific comparisons. It was reported in the literature that the incidence of GDM was reduced in women who underwent BS before pregnancy compared to women with obesity (3, 29). In a study by Lesko et al. (30) it was suggested that the reduction in the incidence of GDM might result from absorptive or metabolic changes following BS. Conversely, studies comparing pregnancies after BS with those in the general population indicated a higher incidence of GDM in the post-BS sample (31). Metabolic changes post-BS were thoroughly described in research by Madsbad et al. (32). Poston et al. reported an increased incidence of GDM in women in the BS group, attributing this to excessive fat accumulation that disrupts glucose metabolism (2). A meta-analysis by Galazis et al. found no discernible differences in GDM between pregnancies following BS and those with matching BMIs before conception. The authors emphasized that diabetes did not arise or worsen due to the surgical condition but was influenced by BMI and the associated hormonal and metabolic environment (10). Our study also demonstrated a significantly higher GDM score in individuals who had undergone BS compared to those who had not. When excluding women with a BMI ≥ 35 kg/m^2^ and BMI 30–34.9 kg/m^2^, the incidence of GDM remained significantly higher in women post-BS in BMI-specific comparisons. No significant differences were detected between the groups in BMI-specific evaluations because the incidence of GDM was also high in women without BS who had a BMI of 30–34.9 kg/m^2^ and BMI ≥ 35 kg/m^2^. However, there are no recommendations for the diagnosis and management of GDM after BS. In our study, it was determined that the need for insulin treatment increased; the need for further studies, including a larger number of cases, cannot be ignored. There are studies in the literature reporting that changes in glucose metabolism are associated with the type of obesity surgery (33), but no evaluations were made regarding this in the present study.

In the literature, there are also studies reporting that the incidence of premature birth increased after BS (2, 10). Some research also showed an elevated incidence that might be partly explained by the requirement for an iatrogenic premature birth and pregnancy problems linked to obesity (2). Some studies found no differences in the iatrogenic preterm birth incidence after BS (33). In our study, the incidence of premature birth was significantly higher in participants who had undergone BS than in those who had not. In this current research, the premature birth incidence in the BMI < 25 kg/m^2^ and BMI 25–29.9 kg/m^2^ groups was significantly higher in participants who had undergone BS than in those who had not in BMI-compatible comparisons. Although the rate of iatrogenic prematurity was not analyzed retrospectively, the incidence of PIH, preeclampsia, and SGA, which are common indications for it, increased significantly in the BS group. Predicting additional risks that BS may cause is challenging because the risk of premature birth increases with maternal age (34). However, despite the lower mean age of participants who had undergone BS in this research, the rate of prematurity was notably higher. Akhter et al.’s meta-analysis reported contrary results and found no differences in post-term birth incidence (14). Investigations into the relationship between BS and C/S incidence have yielded contradictory results (35). Some research identifies BS as a separate risk factor for C/S (31). In our study, the BS group had a considerably higher overall incidence of C/S. When comparing BMI-specific data, no significant differences were detected in C/S rates between participants with and without BS in subgroups with BMI 30–34.9 kg/m^2^ and BMI > 35 kg/m^2^. The small BMI sample size and the limited number of participants involved in C/S make this outcome difficult to interpret, potentially biasing our conclusion.

It was reported by Lapolla et al. that the incidence of C/S increased in women who underwent BS compared to women of normal weight; in the same research, it was also reported that the incidence of C/S decreased when compared to women with obesity who did not undergo BS (36). In the meta-analysis conducted by Akhter et al., the authors showed that the C/S incidence decreased after BS compared to BMI-matched controls (16). Nevertheless, it is quite hard to evaluate the independent effects of BS because obesity and advanced age increase the chance of C/S (37). There are significant inconsistencies in studies regarding the incidence of C/S after BS. A systematic study reported that the rate of C/S increased after BS in comparison with women with obesity with no history of BS (29). A primary study that compared pregnancy outcomes after BS with those in the normal population showed that BS was an independent risk factor for C/S (30). The same research also reported that there were no possible physiological reasons for such effects, stating that this could be a biased outcome. Vrebosch et al. reported that C/S rates were lower after BS (27).

Two more evaluations concluded that more research was necessary to clarify the occurrence of C/S following BS (3, 28). Galazis et al. conducted a meta-analysis and found no significant differences in the rates of C/S among women who had undergone BS, either in the whole group analysis or any of the sub-analyses, as opposed to obese women who had not undergone BS (10). In our study, the C/S rates in the participants who had undergone BS were observed to be significantly higher than in participants who had not, in comparisons made independently of BMI evaluations. The C/S rates were significantly higher in BMI-matched groups, particularly in participants with normal weight and slightly overweight who underwent BS, and no significant differences were observed in participants with obesity and extreme obesity on the basis of C/S rates between the patients who had undergone BS and those who had not.

Women who undergo BS before becoming pregnant have a higher risk of miscarriage for their gestational age compared to pregnant women with obesity (27–29). Although the GDM incidence increased significantly in the group that underwent BS in our study, no significant differences were found in the incidence of LGA. In this study conducted at a tertiary center, we attribute the high rates of GDM and insulin-dependent GDM, as well as the low rate of LGA, to close obstetric follow-up, the correlation work of endocrinologists and dieticians, and patient compliance. In women with BMI > 25 kg/m^2^, the incidence of SGA was significantly higher in the BS group, but no significant difference was found in other BMI groups. Although the relationship between types of surgery and pregnancy outcomes was not analyzed in our study, it was assumed that the relation between malnutrition and SGA was explained, especially following malabsorptive surgeries such as obesity surgery (16). The meta-analysis by Galazis et al. found an approximately 80% rise in the risk of SGA, indicating that the frequency of SGA in the BS group was much higher than in pregnancies matched by pre-pregnancy BMI, in contrast to GDM (10). Our study found that SGA occurred more frequently in women with a history of BS, regardless of BMI measurements. However, the frequency of SGA was significantly higher in the BS group compared to pregnancies with similar pre-pregnancy BMI. Earlier research suggested that the increase in the prevalence of SGA linked to BS was attributed to malnutrition and deficiencies in the microenvironment resulting from the postoperative therapeutic condition (28).

In a study comparing women who had and had not undergone gastric bypass, no difference was reported in the rates of small gestational age. Accordingly, it can be thought that gastric bypass will be beneficial in this respect instead of sleeve gastrectomy (38–40). However, when evaluating the effects of BS, the presence of SGA should be considered together with the presence of large for gestational age due to obesity and type 2 diabetes mellitus (38–40). Our findings did not concur with the previously reported increased risk of stillbirth, which was attributed to fewer fetal movements, potential anomalies, and inadequate diagnosis of fetal macrosomia (2). We found that bariatric patient groups with BMI < 30.0 kg/m^2^ had a considerably higher prevalence of 5th-minute Apgar scores <7. Low Apgar scores were not disclosed in patients who underwent BS in the Kjaer et al. statewide registry-based investigation (35).

Detailed analysis of pregnancies can be considered as the strength of the study. Instead of comparing women with preoperative weight-matched controls (33), we wanted to compare women with a history of BS with BMI-matched controls who had not undergone BS. This factor can be considered another strength of our research. There are also limitations that should be considered when interpreting the results. A registry-based study can only employ variables that have been documented; minor variations in the use of diagnostics between institutions, departments, and individuals cannot be eliminated. The weight gain during pregnancy or possible weight loss following BS was not noted. In addition, only sleeve gastrectomy surgery was performed on the patients in our study, and differences that might arise depending on the type of surgical method emphasized in other studies in the literature could not be evaluated (41, 42). Furthermore, it is impossible to overlook the bias brought about by the limited sample size and comparisons of some variables based solely on BMI.

Important information on how BS affects future pregnancies and deliveries is also provided by this study, which can be helpful in advising women for family planning both before and after BS. The researchers specifically aimed to examine differences in obstetric outcomes between BMI-matched women who had undergone surgery and those who had not because previous BMI data were not available and improvements in obstetric outcomes after BS were already described (20, 21).

Considering this study’s findings and the advantages and disadvantages of BS, we think that this surgery is a recommendable treatment modality for women who are of childbearing age, although its possible risks must also be considered. The authors conclude that pregnancy prognosis is closely correlated with BMI at the time of pregnancy. It was found that patients with and without a history of BS had similar results in terms of pregnancy prognosis and pregnancy complications in the obese and extremely obese groups. According to our results, the risk of hypertensive disorder and GDM was significantly elevated in women with a history of BS. However, no significant differences were detected in the risk of GDM-associated LGA following BS. Close supervision of obstetricians is important to obtain the best result for both the baby and the mother in pregnancies after BS.

## Data Availability

The original contributions presented in the study are included in the article/supplementary material, further inquiries can be directed to the corresponding author.
